# Ertapenem for treatment of osteomyelitis: a case series

**DOI:** 10.1186/1756-0500-4-478

**Published:** 2011-11-02

**Authors:** Neela D Goswami, Melissa D Johnson, Vivian H Chu

**Affiliations:** 1 Department of Medicine, Duke University Medical Center, Durham, NC, USA

## Abstract

**Background:**

Ertapenem is a once-daily broad spectrum carbapenem that is increasingly used to treat polymicrobial osteomyelitis due to diabetic foot and traumatic wound infections. However, limited data exists on ertapenem use for osteomyelitis. This study aimed to characterize outcomes and adverse effects with empiric use of ertapenem for osteomyelitis.

**Findings:**

A total of 112 patients presenting to Duke, Durham Regional or Durham VA Medical Centers with a suspected diagnosis of osteomyelitis and ertapenem use from 11/2001 to 8/2009 were screened, and 12 subjects met inclusion criteria for the study. Mean age was 60 ± 16 years, 68% were female, 75% were Caucasian, and the most common comorbidities included diabetes (58%), peripheral vascular disease (42%), and history of tobacco use (75%). Over half of the patients presented to a primary care clinic or emergency room greater than six months after the onset of clinical symptoms. Bone culture was obtained for diagnostic guidance in only two cases; and surgical intervention was pursued in three cases. Patients received a mean duration of 34.6 ± 7.8 days of therapy, and in three cases, subsequent suppressive oral antibiotics were given. Six (50%) patients met criteria for clinical success, defined as resolution of clinical signs and symptoms of infection such that discontinuation of antibiotics was deemed appropriate at end of ertapenem therapy, without recurrence at one year follow-up. No adverse drug effects were noted.

**Conclusions:**

In this case series of mostly community-acquired, lower extremity osteomyelitis, bone biopsy was infrequent, and an average six-week course of empiric ertapenem was well-tolerated with curative rates of 50% at one year.

## Introduction

Osteomyelitis is a common, costly clinical problem [1]. Challenges to osteomyelitis treatment include difficulty identifying the offending pathogen and the requirement for a long course of intravenous therapy. Osteomyelitis due to diabetic foot and traumatic wound infections are often polymicrobial and require broad-spectrum antibiotics to cover gram-positive, gram-negative, and anaerobic microorganisms. While oral therapy is generally not first-line for serious infections, regimens including beta lactams, quinolones, cephalosporins, and nitroimidazoles have been employed in certain populations [2,3]. Current intravenous options for polymicrobial osteomyelitis therapy include piperacillin/tazobactam, ampicillin/sulbactam, and imipenem. While these intravenous antimicrobials provide excellent broad-spectrum coverage, they require multiple daily doses, which can be inconvenient, particularly in the outpatient setting.

Ertapenem is a broad-spectrum carbapenem with a long half-life permitting once-daily dosing. This antibiotic has activity against most invasive bacterial pathogens except for *methicillin-resistant Staphylococcus aureus *(MRSA), enterococci, *Pseudomonas aeruginosa*, and *Acinetobacter baumannii*. Since ertapenem does not inhibit metabolism of cytochrome P450, it has few drug interactions, and has a side effect profile similar to piperacillin/tazobactam [4]. Given the wide tissue distribution, ease of administration and limited potential for drug interactions, ertapenem may be a good option for treating polymicrobial osteomyelitis. However, there are no randomized control trials and only a few observational studies [5,6] evaluating the use of ertapenem for osteomyelitis. Ertapenem was shown to have similar clinical response rates to piperacillin/tazobactam in a double-blind randomized controlled study of diabetic infections, but patients with osteomyelitis were specifically excluded from that trial [7]. This study aimed to retrospectively analyze data from patients who received ertapenem therapy for osteomyelitis to further characterize outcomes and adverse effects associated with therapy.

## Methods

Patients prescribed ertapenem November 2001 through August 2009 at Duke University, Durham Regional and Durham VA Medical Centers were identified through pharmacy records and cross-matched with patients diagnosed with osteomyelitis during the same hospital stay. Patients 18 years or older with an International Classification of Disease code (ICD-9) discharge diagnosis of osteomyelitis who received at least two weeks of ertapenem were included in the study. Included subjects furthermore had to meet the following clinical criteria for a diagnosis of osteomyelitis: persistent bone pain, erythema, tenderness, or exposure through open wound and one or more of the following: (1) positive culture on bone biopsy; (2) suggestive radiographic findings on CT scan, MRI or bone scan; and (3) operative findings suggestive of infection [8]. Patients were excluded if (1) infection involved prosthetic material in which the material was not removed, (2) an allergy to imipenem or ertapenem was documented, (3) initial infection during studied hospital stay was documented to involve MRSA, *P. aeruginosa*, or enterococci, or (4) vancomycin or another antibiotic was given for over two weeks concurrently with ertapenem.

Demographic and clinical data, drug administration information, and adverse effects were collected on a standardized case report form. The patient's clinical episode of osteomyelitis was classified as an initial episode or recurrence (repeat episode of osteomyelitis at current site). Contiguous osteomyelitis was defined as bone infection associated with wound infection, osteomyelitis following surgery or trauma, or cellulitis at the osteomyelitis site occurring within two weeks prior to diagnosis; hematogenous was defined as any osteomyelitis without a contiguous focus of infection [6]. Osteomyelitis was considered acute if symptoms (bone pain, erythema, tenderness, or exposure through an open wound) were present less than four weeks prior to presentation to a medical provider; otherwise the infection was classifed as chronic. The episode of osteomyelitis was categorized as health-care associated if the patient had transferred to the study hospital from another healthcare facility, received long-term hemodialysis, was hospitalized during the previous 30 days, or was receiving chemotherapy for a malignancy [1]. Outcomes at end of therapy, six weeks, six months, and one year after therapy were assessed through chart review. Cure was defined as resolution of clinical signs and symptoms of infection such that discontinuation of antibiotics was deemed appropriate, with no recurrence at one year follow-up, and failure was defined as the persistence or progression of pain, swelling, erythema, or draining sinus tracts.

The ethical committees that provided approval for the study were the Duke (Pro00019532), Durham VA (MIRB 1465), and Durham Regional (Pro00019532) Medical Centers Institutional Review Boards (IRBs). Given the retrospective chart-based nature of the study, the IRBs at all study sites waived the requirement for informed patient consent. Each patient was assigned a unique study number, free of any patient identifiers. Data was collected retrospectively, confidentially, and was stored in a central, password protected database in a secure location.

## Findings

A total of 112 inpatients at Duke, Durham VA, and Durham Regional Medical Centers were hospitalized with an ICD-9 discharge diagnosis of osteomyelitis and were reported to have received ertapenem during their hospitalization. Of these patients, 12 were excluded because chart review did not confirm a primary clinical diagnosis of osteomyelitis, 12 did not receive at least two weeks of ertapenem, five had positive microbiology data for MRSA involving the current site of osteomyelitis, and 71 concurrently received a second primary antibiotic for at least two weeks along with ertapenem.

In the remaining 12 patients with a diagnosis of osteomyelitis and ertapenem used as main therapy, mean age was 60 (± 16) years, the majority (68%) were female, and 75% patients were Caucasian. The most common comorbidities included diabetes (58%), peripheral vascular disease (42%), and history of tobacco use (75%). Most patients presented to a clinic or emergency room after six months of symptoms from osteomyelitis (see Table 1). Patients were hospitalized a median of 15 days (interquartile range [IQR]: 0-50 days) after their first medical contact. For 83% patients, their osteomyelitis was an initial episode (see Table 2). Over 90% patients had osteomyelitis from a contiguous source, rather than from hematogenous spread, and 33% patients reported preceding trauma. Onset of clinical symptoms of osteomyelitis occurred in a community setting in the majority (92%) of patients, while one patient acquired infection in a local skilled nursing facility. The most commonly affected site of infection was the toe (50%); other affected sites included the calcaneus, malleolus, tibia, and vertebra. MRI was the primary diagnostic imaging modality in 80% of cases where imaging was used; two cases of osteomyelitis were diagnosed on clinical grounds alone. Bone culture was obtained for diagnostic guidance in only two (17%) of cases. Organisms obtained from bone or deep tissue culture included methicillin-susceptible *Staphylococcus aureus*, mixed organisms, *Gemella *spp., and *Klebsiella pneumoniae*. Initial surgical intervention was pursued in 25% of cases, and in one of these, repeat surgery was performed.

**Table 1:** Clinical Course Summary of Osteomyelitis Patients Treated with Ertapenem (N = 12)

Time from Clinical Symptoms to Medical Contact	N (%)
Less than 1 Month	3 (25.0)

1-6 Months	2 (16.7)

Greater than 6 Months	7 (58.3)

**Median Days from Medical Contact to Hospital Admission**	14.5 (IQR: 0-49.5)

**Bone Culture Obtained for Diagnosis**	2 (16.7)

**Initial Surgical Intervention**	3 (25.0)

Repeat Surgery Performed	1 (33.3)

**Mean Duration of Ertapenem Treatment, Days**	34.6 (7.8)

**Clinical Cure Achieved**^£^	6 (50.0)

**Adverse Effects from Ertapenem**^**¥**^	0 (0.0)

**Subsequent Suppressive Oral Antibiotics Prescribed**	3 (25.0)

**Mean Time of Clinical Follow-up after Osteomyelitis Hospitalization**, **Weeks**	115.6 (68.5)

^£^Clinical cure is defined as resolution of clinical signs and symptoms of infection such that discontinuation of antibiotics was deemed appropriate at end of ertapenem therapy, and there was no recurrence of symptoms of osteomyelitis at one year.

**^¥^**Adverse effects evaluated include: rash, nausea, vomiting, diarrhea, seizure, altered mental status, hepatitis, renal insuffiency, and hematologic abnormalities.

**Table 2:** Individual Demographics, Diagnosis, Treatment, and Outcomes of Osteomyelitis Patients Treated with Ertapenem

Patient	Age (yrs)	Race	Gender	**Comorbidities**^**€**^	**Diagnosis**^**¥**^	Trauma-related	DiagnosticMethod	Surgical Intervention	No. Days on Erta	Other Antibiotic	**Outcome at One Year**^**£**^
1	47	White	Male	None	Recurrent chronic contiguous tibial osteomyelitis	Yes	Bone culture	Soft tissue debridement	39	None	F-Infection cleared at end of therapy, but recurred by one year

2	56	White	Female	COPD, DM, Pancreatic cancer	Initial chronic hematogenous vertebral osteomyelitis	Yes	Deep tissue culture	None	39	Suppressive moxifloxacin following ertapenem	F-Did not clear infection at end of therapy, but cleared by one year

3	70	White	Male	CAD, PVD	Initial chronic contiguous tibial osteomyelitis	Yes	Bone culture	Bone debridement	33	None	S-Infection cleared at end of therapy without recurrence at one year

4	41	White	Female	None	Initial acute contiguous toe osteomyelitis	Yes	MRI	None	43	Amoxicillin/clavulanate for 20 days preceding ertapenem	S-Infection cleared at end of therapy without recurrence at one year

5	72	White	Female	PVD, DM, CKD	Initial chronic contiguous toe osteomyelitis	Not known	MRI	None	41	See footnote^1^ Suppressive cephalexin following ertapenem	F- Did not clear infection at end of therapy, but cleared by one year

6	64	Black	Female	PVD, DM	Recurrent chronic contiguous malleolar osteomyelitis	Not known	MRI	None	35	Vancomycin and ampicillin/sulbactam for 6 days preceding ertapenem; suppressive ciprofloxacinclindamycin following ertapenem	F- Did not clear infection at end of therapy, but cleared by one year

7	79	White	Female	None	Initial chronic contiguous calcaneal ostemyelitis	No	Bone scan	None	24	None	S-Infection cleared at end of therapy without recurrence at one year

8	76	White	Female	Neurologic disease	Initial acute contiguous calcaneal osteomyelitis	No	MRI	Amputation	26	Imipenem for 14 days following therapy	S-Infection cleared at end of therapy without recurrence at one year

9	47	White	Female	DM	Initial chronic contiguous toe osteomyelitis	Not known	MRI	None	42	Piperacillin/tazobactam for 9 days prior to ertapenem	S-Infection cleared at end of therapy without recurrence at one year

10	37	White	Male	DM	Initial chronic contiguous toe osteomyelitis	No	MRI	None	31	Ampicillin/sulbactam for 3 days prior to ertapenem	S-Infection cleared at end of therapy without recurrence at one year

11	48	Black	Female	PVD, DM	Initial acute contiguous toe osteomyelitis	No	Plain film	None	20	Vancomycin for 4 days, piperacillin/tazobactam, 3 days, ceftriaxone for 2 days, all prior to ertapenem	F-Did not clear infection at end of therapy, nor by one year

12	86	Black	Male	CAD, PVD, DM	Initial chronic contiguous toe osteomyelitis	No	MRI	None	42	Piperacillin/tazobactam for 5 days prior to ertapenem	F- Did not clear infection at end of therapy, but cleared by one year

^€^Comorbidities include chronic obstructive pulmonary disease (COPD), coronary artery disease (CAD), peripheral vascular disease (PVD), diabetes mellitus (DM), cancer, chronic kidney disease (CKD), liver disease, connective tissue disorder, neurologic disease, human immunodeficiency virus (HIV), and hepatitis C virus (HCV).

^¥^Classification of osteomyelitis included the following definitions: Acute = less than four weeks of symptoms (bone pain, erythema, tenderness, or exposure through open wound) prior to presentation to a medical provider; chronic = greater than or equal to four weeks of symptoms prior to presentation; initial = first episode of osteomyelitis at current site; recurrent = repeat episode of osteomyelitis at current site; contiguous = any associated wound infection following surgery or trauma or cellulitis at the site of osteomyelitis occurring within two weeks prior to diagnosis; hematogenous = any osteomyelitis without a contiguous focus of infection associated with the osteomyelitis site.

^£^S = success, defined as resolution of clinical signs and symptoms of infection such that discontinuation of antibiotics was deemed appropriate at end of ertapenem therapy, and no recurrence at one year; F = failure, any case not meeting criteria for success.

^1^Vancomycin for five days, ciprofloxacin for one day, metronidazole for two days, ceftazadime for four days, and imipenem for two days, all preceding ertapenem, and six days of clindamycin concurrently with ertapenem.

In total, patients received a mean duration of 35 ± 8 days of ertapenem. Subsequent suppressive oral antibiotics were given in three cases. In one case, a patient with vertebral osteomyelitis had recurrence of back pain and new *Enterococcus gallinarum *bacteremia within six months of intravenous therapy; she then received moxifloxacin 400 mg orally daily; and by one year she was considered cured and antibiotics were discontinued (see Table 2). In the second case, a diabetic patient with osteomyelitis of the proximal phalanges continued to have symptoms at the end of intravenous therapy and was given cephalexin 1 g orally every 8 hours; clinical success was achieved before 6 months, and antibiotics were stopped. In the third case, the patient was a diabetic with osteomyelitis of the lateral malleolus, and with continued signs of infection at the end of therapy, she was given ciprofloxacin 500 mg twice daily and clindamycin 150 mg orally three times daily for three months; she subsequently remained free of infection at 6 months and at one year. In the total series, six (50%) had clinical success at one year after therapy.

There were two cases of recurrence at one year. The first was a 47 year-old man with a remote tibial fracture complicated by previous MRSA infection treated with vancomycin who subsequently developed new drainage from the tibia during the study hospitalization and underwent debridement; cultures grew only *Gemella spp*., and he then received ertapenem for 39 days. He was considered a clinical success at end of therapy, six weeks, and six months afterwards. However, at one year he developed new infection with tissue cultures growing MRSA.

The second clinical recurrence occurred in a 48 year-old woman with diabetes and peripheral vascular disease admitted with contiguous osteomyelitis from an infected diabetic foot ulcer. She received 20 days of empiric ertapenem with no surgical intervention or subsequent antibiotics. After three weeks of the studied hospitalization, she developed bacteremia with MRSA and *Bacteroides fragilis *attributed to acute osteomyelitis at the same site. Despite various antibiotic regimens, she had subsequent recurrent flares over one year later.

## Discussion

In this series, we present 12 cases of osteomyelitis treated with ertapenem as the primary antimicrobial. We note a success rate of 50% among patients who received an average of six weeks of ertapenem. This success rate is similar to outcomes found in other retrospective studies of conservatively treated contiguous osteomyelitis [2,9]. In a case series evaluating use of ertapenem for gram-negative infections, cure rate was found to be 64%, but only five patients with osteomyelitis were included in that study [10].

Three patients in our series went on to receive suppressive oral antibiotics;, all three were ultimately free of symptoms by one year, suggesting that ertapenem may require adjunctive therapy for clinical cure in complex cases. In total, only two patients in the series continued to have symptoms related to osteomyelitis at one year after therapy. In both cases, recurrent infections were attributed to MRSA, an organism known to be resistant to ertapenem.

While traditional teaching for osteomyelitis is to pursue bone biopsy and culture for optimal antibiotic choice and management [11,12], the absence of intervention in this series is striking, with only two patients undergoing bone biopsy. Other studies have shown this is not uncommon in cases where orthopedists and interventional radiologists are not engaged in the patient's care [13]. Whether improving the use of bone biopsy for diagnostic purposes would improve patient outcomes is unclear from our data and warrants further study. Concomitant surgical intervention (which occurred in three patients in our series) has also been shown to improve patient outcomes in serious diabetic foot infections [14], and may have changed overall cure rates if performed more consistently in our patients.

This study is retrospective in nature and has a limited number of study subjects treated with ertapenem alone. In addition, our results are subject to selection bias since patients with less severe cases of osteomyelitis may have been chosen for monotherapy with ertapenem rather than combination therapy. Nevertheless, this series demonstrates efficacy and a favorable safety profile for ertapenem use in lower extremity osteomyelitis. The study demonstrates curative rates of 50%, and clinical resolution of infection approaching over 80% at one year, specifically when surgical intervention and suppressive oral antibiotics are concurrently employed and MRSA is unlikely to be a pathogen.

## Competing interests

MDJ and VC have received funds for research from Merck&Co. All other authors: none to declare. Merck&Co. had no role in the design, execution, or analysis of this study. No medical writer was used.

## Authors' contributions

NG, MDJ and VC conceived of the study and participated in its design and coordination. NG performed the statistical analysis and drafted the manuscript. All authors read and approved the final manuscript.

## Funding

This work was supported by a Merck investigator-initiated grant (VHC).
